# Y-shape osteotomy combined with subtalar arthrodesis for calcaneus malunion: a retrospective study

**DOI:** 10.1186/s13018-022-03413-w

**Published:** 2022-12-07

**Authors:** Wenbo Bai, Yongzhan Zhu, Junkui Xu, Jingqi Liang, Jun Lu

**Affiliations:** 1 Department of Foot and Ankle Surgery, Foshan Hospital of Traditional Chinese Medicine, Foshan, 528000 Guangdong People’s Republic of China; 2 Foot and Ankle Surgery Department, Honghui Hospital of Xi’an Jiaotong University, Xi’an, 710000 Shaanxi People’s Republic of China

**Keywords:** Calcaneus malunion, Y-shape osteotomy, Subtalar arthrodesis, Treatment

## Abstract

**Background:**

This retrospective study aimed to introduce a novel method for simultaneous Y-shape osteotomy combined with subtalar arthrodesis for calcaneus malunion and to evaluate the feasibility of this method.

**Methods:**

We retrospectively analysed the clinical and imaging data of 11 patients with calcaneus malunion treated using *Y*-shape osteotomy and subtalar arthrodesis who were admitted to our hospital from June 2018 to October 2020. The patients included 9 males and 2 females aged from 24 to 69 years old, with an average age of 42.18 years. The clinical and radiological results were assessed with the Visual Analogue Scale (VAS) pain score and American Orthopaedic Foot and Ankle Society (AOFAS) hindfoot score. In addition, functional recovery and general quality of life were evaluated using the 12-Item Short-Form Survey (SF-12).

**Results:**

All radiological parameters were significantly improved at the last follow-up, with increases of 14.37°, 9.18°, and 4.51 mm in the Böhler’s angle, calcaneal pitch angle, and talocalcaneal height, respectively, and decreases of 6.39 mm and 6.18° in the calcaneal width and Hindfoot alignment angle (*p* < 0.05). The mean AOFAS and VAS scores at the last follow-up improved compared with those preoperatively, from 34.18 ± 9.53 to 84.18 ± 11.59 and from 6.90 ± 1.22 to 1.90 ± 1.13, respectively (*p* < 0.05). The SF-12 physical and mental health scores were 49.65 ± 6.84 and 52.68 ± 7.88, respectively. Furthermore, the early postoperative complications included skin necrosis in one and sural neuralgia in one patient, and the late postoperative complication included ankle pain in one patient. No other complications, such as implant discomforts, malunion, nonunion and re-fracture, were presented.

**Conclusion:**

These results indicate that Y-shape osteotomy combined with subtalar arthrodesis is an effective new method for the treatment of calcaneal malunion. Advantages include improvement of the anatomic shape of the calcaneus and union rates for subtalar arthrodesis.

## Introduction

Calcaneus fracture is very common in the clinic, accounting for approximately 2% of systemic fractures and 60% of all tarsal fractures, usually as a result of high-energy trauma, such as traffic accidents or falling [1]. Due to the complexity of the hindfoot structure and high-energy trauma, inappropriate conservative or surgical treatment often leads to calcaneal malunion in the later stage and even severe traumatic subtalar arthritis. Calcaneal malunion is often accompanied by subtalar joint depression, arch collapse, widening of the calcaneus, and varus/valgus deformity of the hindfoot, which are important causes of severe pain and disability in patients [2].

Sanders et al. [3] reviewed the long-term results (10–20 years, mean 15.2, after ORIF of calcaneal fracture), found that one in three patients required fusion, despite anatomic reduction in 95% of cases. Csizy et al. [4] reviewing the data of a previous RCT from the same group, found that nonoperative management resulted in more frequent need for subtalar arthrodesis. In particular, men involved in heavy labour, receiving workers' compensation and with Bohler’s angle less than 0° are more likely to undergo secondary subtalar fusion if initially treated non-operatively [5].We believe that for high-energy trauma calcaneal fractures, especially Sanders type III or above, operative management may be a better option than non-operative management, and that despite initial anatomic reduction, subtalar arthritis remains an unavoidable practical problem in long-term follow-up.

Subtalar arthrodesis is the best treatment for traumatic subtalar arthritis, but the complex pathological features brought by calcaneal malunion should not be ignored.

At present, depending on the type of deformity, many authors have advocated different reconstructive strategies. The mainstream techniques for calcaneal malunions include lateral wall decompression, in situ subtalar arthrodesis, arthroscopic subtalar arthrodesis, distraction subtalar arthrodesis, corrective calcaneal osteotomy with arthrodesis, and triple arthrodesis, among which distraction subtalar arthrodesis and in situ subtalar arthrodesis are the most popular [6–8].The goal of these measures is correction of the deformity and relief from the pain by osteotomy and subtalar arthrodesis. But there are still shortcomings, such as nonunion at the fusion site and limited correction of the calcaneal deformity. To solve the complex pathological problems caused by calcaneal malunion, expand the surgical techniques of subtalar arthrodesis, and minimize the complications of subtalar arthrodesis, we designed a new technique to maximize the original anatomy of the calcaneus and improve bone healing at the fusion site of the subtalar joint by extraarticular Y-shaped osteotomy of the calcaneus. Therefore, the purpose of this retrospective study is to introduce a new technique of calcaneal osteotomy and subtalar arthrodesis with broader indications and to evaluate the clinical and outcomes and impact of this new technique on complex malunion of the calcaneus with severe subtalar arthritis.

## Materials and methods

### Study design and patient population

This study was approved by the Ethics Committee of Honghui Hospital Affiliated to Xi'an Jiaotong University (NO. 202111002), and all patients provided written informed consent to participate in the study and for all operative procedures performed.

The inclusion criteria were as follows: calcaneus malunion > 6 months, last follow*-*up > 12 months, a calcaneus malunion of type II or III according to the Stephens–Sanders classification, extensive subtalar joint degeneration on imaging, significantly collapsed arches. The exclusion criteria were as follows: bilateral calcaneal fractures, poor skin healing at the original incision site, diabetes with blood glucose that was not well controlled, history of systemic osteoarticular diseases such as rheumatoid arthritis and Charcot’s joint, and incomplete follow-up data. We retrospectively analysed 11 patients that met the inclusion and exclusion criteria from June 2018 to October 2020. All patients underwent Y-shaped calcaneal osteotomy and subtalar arthrodesis, and the operations were performed by the same physician. The patient information is shown in Table 1.

**Table 1:** Patient information

Case	Age	Sex	Injured sites	Case of injury	Initial treatment	Time from initial to fusion (months)	Stephens–Sanders	Follow-up (months)
1	24	M	L	Fall	ORIF	36	II	12
2	34	M	L	Fall	Cast	27	II	12
3	34	M	L	Vehicle accident	ORIF	19	III	21
4	47	M	L	Fall	PRIF	9	II	15
5	50	W	L	Exercise	Cast	24	III	16
6	46	M	R	Fall	PRIF	19	II	12
7	69	M	L	Fall	Cast	9	III	13
8	35	M	L	Exercise	PRIF	10	III	18
9	45	M	R	Fall	ORIF	15	II	19
10	55	W	L	Fall	ORIF	20	II	20
11	25	M	R	Fall from stairs	Cast	25	III	23

*ORIF* open reduction internal fixation; *PRIF* percutaneous reduction internal fixation

### Surgical technique

**Fig. 1:**
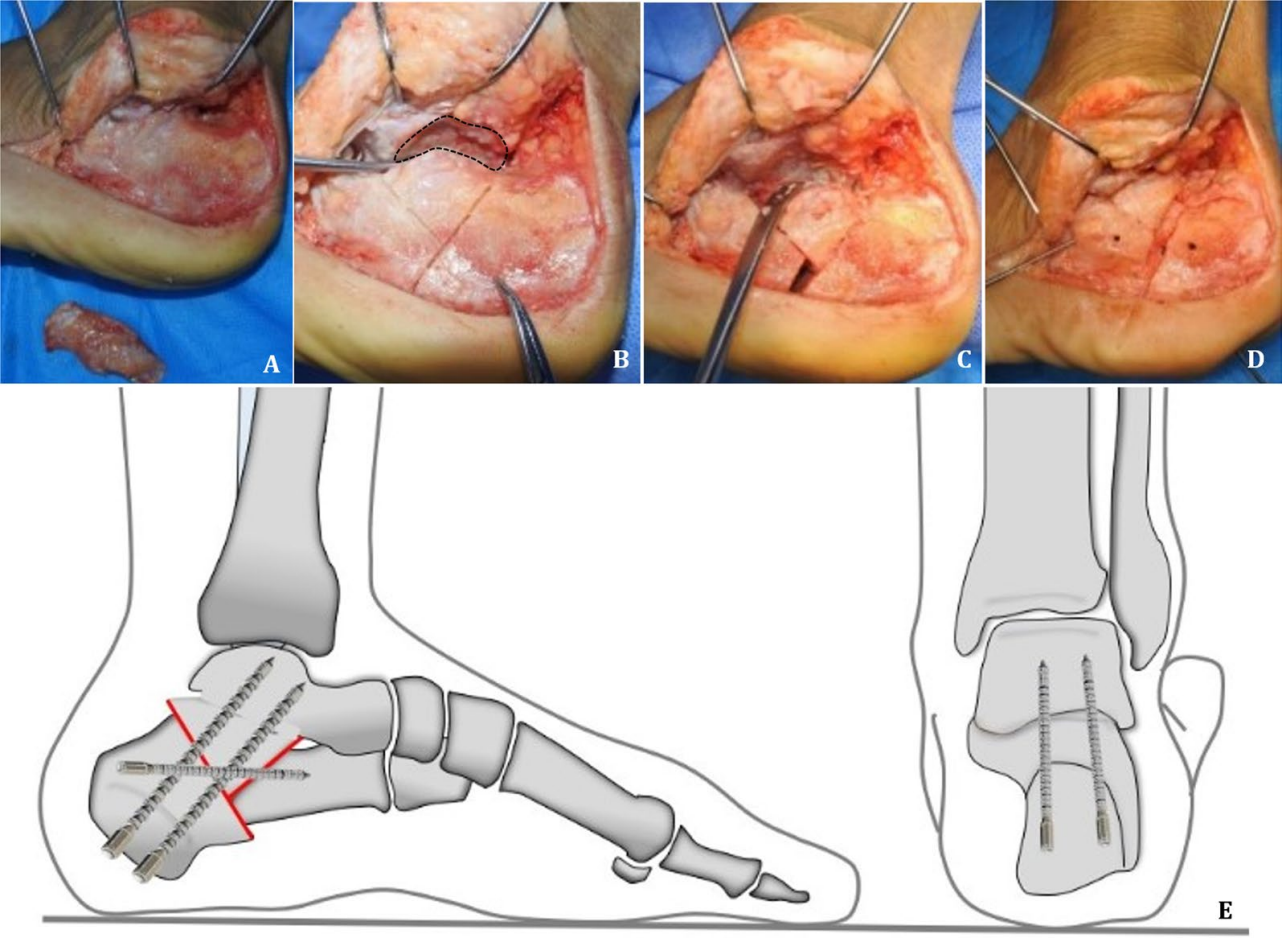
Illustrations of the surgical procedures and technique. **A** Resection of the lateral wall of the bulged calcaneus as a backup bone graft. **B, C** The subtalar articular cartilage is severely damaged, and a Y-shaped osteotomy is performed. **D** Restoring the anatomy of the calcaneus. **E** Schematic diagram of the Y-shaped calcaneal osteotomy and subtalar arthrodesis

The patients were placed on a radiolucent operating table in the contralateral decubitus position of the injured limb, and they were placed under general anaesthesia and had epidural anaesthesia. An L-shaped extended lateral incision was made to expose the subtalar joints and fractured fragments. Three 2.0 mm Kirschner wires were used to drill into the distal fibula, talus neck, and cuboid through the incision, and the full-thickness flap was lifted by bending the Kirschner wires to expose the lateral wall of the calcaneus and the subtalar joint. The lateral wall of the calcaneus was repaired with a pendulum saw, and the remaining bone fragments were saved (Fig. 1A). At 1 cm from the posterior edge of the posterior articular surface of the calcaneus, an oblique osteotomy was made to the plantar side with a pendulum saw. The osteotomy surface was 90° with the lateral wall of the calcaneus, and the osteotomy line was approximately 45° with the plantar surface, which was the first osteotomy line. The lowest point of the Gissane’s angle of the calcaneus, which was perpendicular to the first osteotomy line, was found and was the second osteotomy line, and the two osteotomy lines intersected with a "Y" shape (Fig. 1B). Attention was given not to cut off the medial wall of the calcaneus when performing osteotomy with a pendulum saw. To prevent injury to the medial calcaneal neurovascular bundle, the medial wall was gently interrupted with an osteotome. First, the middle osteotomy block was adjusted, not only to clearly expose the subtalar articular surface so that the damaged cartilage surface could be cleaned more thoroughly but also to restore the matching degree of subtalar joint fusion (Fig. 1C). However, it should be noted that we use a 1.2-mm K-wire for temporary fixation. One end of the K-wire should be placed in the middle osteotomy block, and the other end should be pierced through the dorsa of the midfoot to prevent the slippage of the posterior bone block from being blocked and facilitate the next reduction. Second, the anterior and posterior calcaneus fragments were adjusted to restore the calcaneal height and valgus/valgus deformity under direct vision. Finally, the residual bone of the lateral wall of the calcaneus was implanted at the end of the osteotomy to increase the length of the calcaneus (Fig. 1D). Two 5.2 mm cannulated screws were chosen to fix the subtalar joint, and one 5.2 mm cannulated screw or plate was chosen to fix the calcaneal osteotomy end. Intraoperative fluoroscopy showed that the screw position was satisfactory, and the calcaneal anatomical morphology was restored. After washing the wound with saline, a drainage tube was placed, and the incision was sutured in layers (Fig. 1E).

### Postoperative management

After cold compression for 24 h, routine second-generation cephalosporin antibiotics were administered postoperatively for 48 h to prevent infection, and the drainage tube was removed. Passive and active motion exercises of the metatarsophalangeal joint without weight bearing were initiated the day after surgery. The wounds healed without infection, and all stitches were removed after 2 weeks. In addition, non-weight-bearing functional training, such as cycling, was encouraged while the patient was in bed. The patient would apply a removable boot for 6–8 weeks and remain non-weight bearing. At 6 weeks, the patient was seen in the office and radiographs were obtained. And they may begin to walk with the walking boot, depending upon their level of discomfort. And partial weight-bearing (20–30 kg) was allowed as soon as the patient tolerated it. Stationary bicycle training could be carried out, and resistance training could be gradually increased after 8–10 weeks. Gradual weight-bearing exercises were started, and X-rays taken 12 weeks after the operation showed complete fracture healing and enabled clearance for bearing weight.

### Clinical and radiographic assessment

X-ray and CT scans were obtained in all patients before surgery, after the operation and at the latest follow-up. The patients underwent clinical assessment and were analysed. The functional assessment was performed preoperatively and at the latest follow-up clinic visit based on the American Orthopaedic Foot and Ankle Society (AOFAS), visual analogue score (VAS) and SF-12 [9]. Moreover, the mental component score (MCS) and physical component score (PCS) were calculated from the SF-12 based on Ware et al.’s Manual [10]. The Böhler angle, pitch angle, hindfoot alignment angle, calcaneal width and talocalcaneal height were recorded preoperatively and at the final follow-up. The outcome evaluation consisted of a clinical examination and a radiographic evaluation, which was carried out by two orthopaedic surgeons who were not involved in the operation.

### Statistical analysis

Statistical analyses were performed with SPSS 25.0 software. A Shapiro‒Wilk test for normality was conducted on all continuous data, and continuous variables were presented as the mean ± standard deviation. If the results revealed a normal data distribution, the paired sample t test was used for intragroup comparison. If the data were abnormally distributed, the nonparametric Wilcoxon signed-rank test was used for intragroup comparisons. The level of significance was set at 0.05 for all analyses.

## Results

### Clinical outcomes

Compared to the preoperative values, the AOFAS, VAS, SF12-PCS and SF12-MCS scores improved at the last follow-up (all, *p* < 0.05). Meanwhile, the mean AOFAS score increase was 50; the mean VAS score decrease was 5. The SF12-PCS and SF12-MCS scores were significantly improved at the last follow-up, with increases of 20.55 and 15.98, respectively (Table 2).

**Table 2:** Preoperative and last follow-up clinical outcomes (*x* ± *s*, *n* = 11)

Item	Preoperative	Last follow-up	*t/Z* test	*p value*
VAS	6.90 ± 1.22	1.90 ± 1.13	*Z* = − 2.947	0.003
AOFAS	34.18 ± 9.53	84.18 ± 11.59	*t* = − 10.385	0.000
SF12-PCS	29.10 ± 4.46	49.65 ± 6.84	*t* = − 9.159	0.000
SF12-MCS	36.70 ± 6.12	52.68 ± 7.88	*t* = − 8.253	0.000

### Radiographic outcomes

**Fig. 2:**
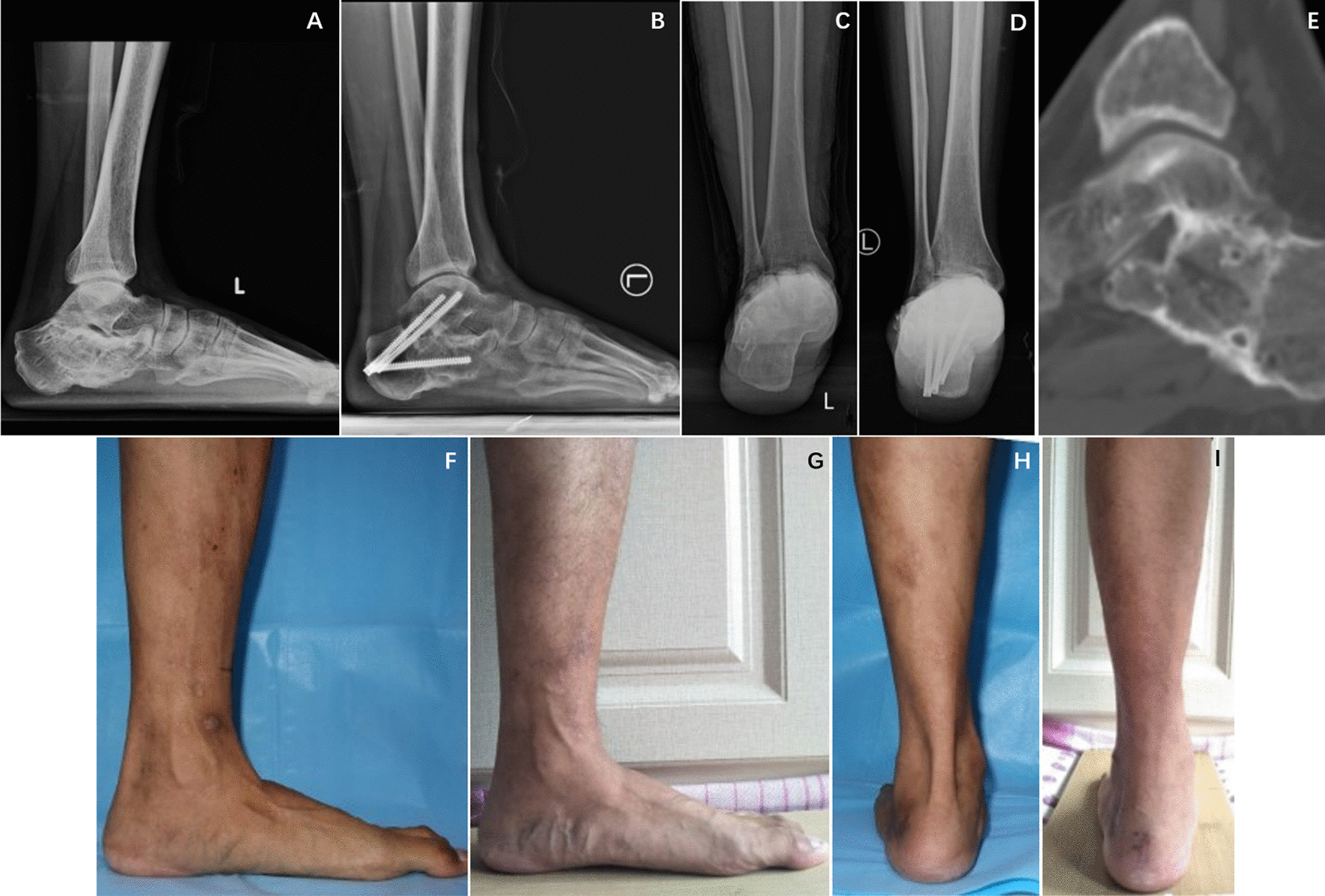
A 35-year-old male patient with left calcaneal malunion for 14 months was treated with Y-shaped calcaneal osteotomy and subtalar arthrodesis. **A**, **B** At the last follow-up, the calcaneal pitch angle, Böhler angle and talocalcaneal height were significantly improved compared with the values before the operation, and the osteotomy end and subtalar joint were osseously healed. **C**, **D** At the last follow-up, the calcaneal width and Hindfoot alignment angle were significantly improved compared with the values before the operation. **E** CT sagittal scan at the last follow-up showed bony union of the fusion site. **F-I** Comparison of external photographs of the patients before surgery and at the last follow-up

Compared to the preoperative values, the calcaneal width and Hindfoot alignment angle had decreased by the last follow-up, whereas the Böhler angle, Calcaneal Pitch angle and Talocalcaneal height had increased by the last follow-up (all, *p* < 0.05). A representative case is shown in Fig. 2. The mean Böhler angle increase was 14.37°; the mean calcaneal pitch angle increase was 9.18°; the mean talocalcaneal height increase was 4.51 mm; the mean calcaneal width decrease was 6.35 mm; and the mean hindfoot alignment angle decrease was 5.18° (Table 3).

**Table 3:** Preoperative and last follow-up radiographic measurements (*x* ± *s*, *n* = 11)

Item	Preoperative	Last follow-up	*t test*	*p value*
Böhler’s angle (°)	11.72 ± 8.31	26.09 ± 3.70	− 5.990	0.000
Calcaneal Pitch angle (°)	13.72 ± 4.56	22.90 ± 4.25	− 8.661	0.000
Hindfoot alignment angle (°)	9.45 ± 2.16	3.27 ± 1.67	9.597	0.000
Calcaneal width (mm)	44.15 ± 4.18	37.76 ± 3.50	8.162	0.000
Talocalcaneal height (mm)	73.74 ± 4.19	78.25 ± 3.71	− 7.263	0.000

### Complications

The early postoperative complications included skin incision necrosis (1 case, 9.0%) and sural neuralgia (1 case, 9.0%). Of the patients, one patient had skin incision necrosis that resolved with dressing changes. The other patient had sural nerve injury and was administered neurotrophic drugs. At the last follow-up, the patient complained that there was still a sense of numbness in the dorsal foot during the aggravation of daily activities, but it was significantly less than that before the operation. The late postoperative complications (1 year or more) included ankle pain (1 case, 9.0%), which intensified after activity. However, the patient could almost resume his daily activities without crutches. We suggested strengthening his rehabilitation program to alleviate the pain.

## Discussion

The pathological anatomy of calcaneal malunion is complex and diverse, but there are some commonalities. First, under high-energy trauma, as little as 2 mm of displacement along the articular surface of the subtalar joint can greatly alter the contact pressure, while conservative, non-anatomical reduction and irreversible damage to the cartilage by primary violence may further develop into posttraumatic arthrosis [6]. Severe subtalar arthritis can cause pain on the lateral side of the calcaneus and erode the remaining subtalar cartilage, seriously affecting the patient's quality of life. It is one of the common complications of calcaneal malunion. Second, due to axial stress, the normal height of the calcaneus is easily lost, which may lead to a decrease in the talocalcaneal angle and lever arm for the gastrocnemius-soleus complex, resulting in reduced push-off power during the patient’s gait, traumatic flatfoot and tibiotalar impingement. Meanwhile, the increase in the calcaneal width is also one of the pathological deformities of calcaneal malunion because it can cause lateral impingement and peroneal tendinopathy or displacement. Finally, the ultimate constellation of symptoms that patients present with will be dependent on the combination of increased calcaneal width, a decreased calcaneal height, hindfoot varus and posttraumatic arthrosis. For Stephens–Sanders II–III calcaneal malunion, subtalar arthrodesis is the gold standard for the treatment of arthritis of the subtalar joint [11]. However, if the talocalcaneal height, calcaneal width and hindfoot alignment angle cannot be restored, the malunion can cause all kinds of complex pathological problems that cannot be solved. Gavlik et al. [12] believe that correcting the Böhler angle, Gissane angle, calcaneal width and calcaneal gravity line of the calcaneus are the criteria for accurate reduction.

Determining the efficacy of different surgical treatments can help doctors formulate different treatment plans according to the patient's condition. Agren et al. [13] performed in situ subtalar arthrodesis in 29 patients. During a long-term follow-up of 7 to 28 years, X-ray and CT scans showed that there were severe residual deformities in the affected limbs, and they concluded that in situ subtalar arthrodesis may not be the best approach for the treatment of severe calcaneal malunion. Some scholars have combined Dwyer osteotomy to solve the problem of angular deformity of the hindfoot after in situ subtalar arthrodesis, but this technique still cannot restore the calcaneal height and collapse of the subtalar joints [14]. We believe that in situ subtalar arthrodesis is suitable for patients with mild calcaneal malunion and subtalar arthritis, and the compensation of the midfoot joint may compensate for various pathological characteristics caused by mild malunion. However, for the malunion of Stephens–Sanders type III or when there is a calcaneal height loss, the hindfoot deformity, traumatic flatfoot deformity and bulge of the lateral wall of the calcaneus were ignored. Our Y-shaped osteotomy technique effectively restores the calcaneal height and the hindfoot alignment by adjusting the posterior calcaneal tubercle fragments, is used to resect the lateral wall of the calcaneus bulge, and can be used to reconstruct the anatomical morphology of the hindfoot. The radiographic parameters of the Böhler angle, pitch angle, calcaneal width, talocalcaneal height and hindfoot alignment angle were significantly improved compared with the values before the operation at the last follow-up, and the patients had no problems with anterior ankle impingement, peroneal tendinitis, contracture of the triceps surae muscle or other problems, which was in line with the goal of Gavlik [12] in the treatment of calcaneal malunion.

Carr et al. [15] first advocated distraction subtalar arthrodesis in 1988. Ideally, the size, shape and position of the bone graft should be selected to fill the subtalar joint and solve the problem of calcaneal malunion, which has been recognized and developed by many scholars. However, we believe that distraction subtalar arthrodesis has limited strength in correcting calcaneal deformities, and it easily causes varus hindfoot deformities. In addition, the use of allogeneic bone undoubtedly increases the economic burden on patients. The fusion rate of allogeneic bone for the subtalar joint is worrisome. On the one hand, when Trnka et al. [16] used allograft bone, 4/5 patients developed nonunion at the fusion site. On the other hand, taking the iliac bone or a medial tibial bone graft increases the secondary trauma, causing pain in the donor site in as high as 29% to 38% of the patients [17]. Myerson et al. [18] pointed out that Carr et al. had poor results in 7 out of 14 cases after fusion, mainly due to the decrease in the calcaneal height caused by bone graft resorption. Although our Y-shaped osteotomy technique does not fill the subtalar joint with bone graft, it does not cause loss of the calcaneal height, which better makes up for the complications caused by bone grafting in the subtalar joint, such as varus deformity, bone graft resorption, nonunion and rejection. The correction of Y-shaped osteotomy is more obvious, and at the same time, it also reduces the economic burden on inpatients. In this study, there was 1 case of sustentaculum tali swelling and pain at the last follow-up, but all the subtalar joints showed osseous fusion on CT.

Huang et al. [19] performed calcaneal metatarsal slip osteotomy combined with subtalar arthrodesis to treat calcaneal malunion, and this technique is similar to our osteotomy. However, it cannot make the cleaned calcaneal articular surface directly and full contact, and it is easy to cause a loss of the calcaneal length. In addition, due to the limitation of the fibrous scar tissue between the calcaneocuboid joint and talocalcaneal interosseous during the operation, it is more difficult to restore the good match of the subtalar joint by extra-articular osteotomy after the calcaneal malunion. Second, to ensure fusion, bone grafts are often required to fill the subtalar joint space, and the resorption of bone grafts may lead to further fusion failures, such as incomplete fusion or nonunion. Therefore, our Y-shaped osteotomy technique implants the lateral wall bone block in the calcaneal body to alleviate the degree of calcaneal length loss. During the operation, the position of the osteotomy block in the middle of the calcaneus is relatively flexible, and it can be used to clearly clean the subtalar cartilage and fully match the contact area of the subtalar joint surface, thereby improving the subtalar joint bony fusion rate and avoiding the problem of bone grafting in the sensitive subtalar joint space. In this study, Y-shaped calcaneal osteotomy and subtalar arthrodesis effectively relieved the clinical symptoms of patients with calcaneal malunion. At the last follow-up, the AOFAS score was improved by an average of 50 points compared with the preoperative level. According to the standard scoring algorithm (USA) [10], the SF12-MCS and PCS were compared to the general population norms, with no statistically significant differences (*t* = 1.131, *P* = 0.284; *t* = 0.168, *p* = 0.870), suggesting that the study population in this group recovered to ideal physical and mental health. Subjectively, the patients were very satisfied with the fusion results, all patients returned to their daily life and work, and 4 patients could even perform high-intensity mountain climbing exercises.

Fletcher et al. [20] systematically reviewed subtalar arthrodesis, included 467 patients (492 feet) and found that nonunion (30/492 feet, 6.1%) and hardware prominence (30/492 feet, 6.1%) accounted for more than 1/3 of all complications (60/161, 37.3%). The follow-up treatment of nonunion complications is very difficult, so it is very important to avoid nonunion or bone graft resorption at the fusion site in subtalar arthrodesis. In this study, there were no problems, such as bone nonunion or hardware prominence, during the last follow-up. We found that the early postoperative complications included skin incision necrosis (1/11 feet, 9.0%) and sural neuralgia (1/11 feet, 9.0%), and the late postoperative complication was ankle pain (1/11 feet, 9.0%), which accounted for more than 1/4 of all complications (3/11 feet, 27.2%) but did not significantly affect the patients' daily life. Among the patients, 1 patient developed skin edge necrosis of the incision 1 week after the operation, which may have been the patient's second operation, and the scar tissue vitality of the original "L" incision was poor. Alternatively, this may have been caused by the calcaneus undergoing plantar slip osteotomy during the operation and the tension of the skin flap at the incision was too large. Therefore, we suggest that in the secondary operation of the calcaneus, the tension-reducing incision and the vacuum sealing drainage technique can be used together. In this study, 6 cases of Stephens–Sanders type II and 5 cases of Stephens–Sanders type III patients. In particular in patients with long-standing, severe deformities, there may be Achilles tendon retraction caused by walking posture, so we will design to lengthen the Achilles tendon before the operation.

Wound healing problems and infections are a frequent complication, reported around 20–45% of patients when extensile lateral approach has been used [5, 21].Sinus tarsi approach and minimally invasive surgery may be viable alternatives, offering similar results with fewer wound complications, but most of the available studies are of low to moderate quality [21]. We also believe that sinus tarsi approach or minimally invasive can be less risky for wound complications, but our Y-shaped osteotomy technique is not always suitable. Because it involves not only the cleaning of the articular surface of the calcaneus, but also a wider range of osteotomy and adjustment of the force line. For patients with severe calcaneal malunion, the sinus tarsi approach cannot fully expose the calcaneal body, and it is difficult to judge the reduction quality of calcaneal osteotomy during the operation, which relatively increases the operation time and technical difficulty, and may further increase the risk of postoperative infection. Correspondingly, the 11 patients with calcaneal malunion included in our group all used the L-shaped extended lateral incision, and only 1 case (1/11 feet, 9.0%) of skin incision necrosis occurred, which is far lower than the rate reported in the literature. And our findings support the management of calcaneal malunion through the L-shaped extended lateral incision technique. Meanwhile, the Y-shaped osteotomy requires sliding the posterior calcaneal fragment to the plantar side, which is often limited by the traction of the Achilles tendon. Therefore, additional lengthening of the Achilles tendon is also performed during the operation, which is performed as a percutaneous Z-plasty. The postoperative patients underwent a series of guided rehabilitation, and no limitation of foot dorsiflexion was found in the last follow-up.

Therefore, we believe that Y-shaped osteotomy and subtalar arthrodesis may have the following advantages: (1) by sliding the posterior osteotomy to the plantar side, problems such as calcaneal height and traumatic flatfoot are improved. (2) By internal/external sliding of the posterior calcaneal osteotomy block, the angulation deformity of the calcaneal axis is improved, and the lower limb force line is better restored. (3) Through the lateral calcaneal incision, it is convenient to fully remove the lateral wall of the calcaneal bulge so that the calcaneal width can be restored to normal, and the peroneal tendinopathy caused by subfibular impingement can be solved. At the same time, the lateral wall bone fragment can be implanted into the calcaneus "Y"-shaped osteotomy, which reduces the loss of the calcaneal length. (4) By adjusting the middle osteotomy block, not only is the contact of the collapsed subtalar articular surface more sufficient and matched, additional allograft filling is unnecessary. The subtalar articular surface can also be clearly exposed during the operation, which is convenient for the surgeon to thoroughly clean the damaged subtalar cartilage. Both of these abilities further improve the bony fusion rate of the subtalar joint. However, the method is accompanied by obvious shortcomings: (1) Although the calcaneus length problem is alleviated by filling the calcaneal body with lateral wall fragments, the calcaneal length may not be fully restored. (2) Due to Y-shaped osteotomy, there is a large amount of skin tension, and complications such as delayed wound healing and infection can easily occur.

There are several limitations to this study. First, this was a single-centre retrospective case series with inherent limitations of the study design, further prospective studies are needed. Second, the number of cases was limited, and the follow-up duration was short, which could have an impact on the results. Third, our study did not involve a control group. It may be interesting to compare the clinical and radiologic changes following our technique versus the changes seen following distraction subtalar arthrodesis.

## Conclusion

In summary, Y-shaped calcaneal osteotomy and subtalar arthrodesis have a significant clinical effect on calcaneal malunion and can effectively restore the calcaneal height, width and alignment. At the same time, the matching degree of the subtalar joint is adjusted without bone grafting, which effectively reduces the formation of nonunion after subtalar joint arthrodesis and adds a new surgical method for the treatment of complex calcaneal malunion.

## Data Availability

The datasets for the present study are available from the corresponding author upon reasonable request.
